# Grain under pressure: Harnessing biochemical pathways to beat drought and heat in wheat

**DOI:** 10.1111/tpj.70253

**Published:** 2025-06-18

**Authors:** Itsuhiro Ko, Tyler Chapman, Taras Nazarov, Ruth Uwugiaren, Andrei Smertenko, Niharika Nonavinakere Chandrakanth, Dylan Oates

**Affiliations:** ^1^ Department of Plant Pathology Washington State University Pullman Washington 99164 USA; ^2^ Program of Molecular Plant Sciences Washington State University Pullman Washington 99164 USA; ^3^ Institute of Biological Chemistry Washington State University Pullman Washington 99164 USA; ^4^ Department of Crop and Soil Sciences Washington State University Pullman Washington 99164 USA

**Keywords:** wheat, grain development, abiotic stress, heat, drought, starch biosynthesis, biochemical pathways

## Abstract

Erratic climate patterns represent a remarkable challenge to global food security, particularly affecting staple cereal crops of which wheat (*Triticum aestivum*) plays a critical role in annual agricultural production globally. It has been shown that over the last four decades, wheat cultivation has faced an escalating vulnerability to a variety of abiotic stresses, including heat and drought. These stressors not only decrease overall yield but also compromise grain quality, leading to reduced soluble starch content, higher protein content, altered grain texture, diminished end‐use quality, and various other undesirable changes. With climate change projections indicating an intensification and higher frequency of heat and drought conditions in the future, urgent action is needed to develop resilient wheat varieties. Achieving this goal relies on a comprehensive understanding of the molecular responses to environmental shifts during successive stages of reproduction. Here we discuss three types of critical biochemical pathways responsible for sustaining starch biosynthesis in both source and sink tissues under adverse environmental conditions during grain development: (i) signaling network and cross‐talk between ABA and SnRK pathways; (ii) transcriptional changes of the enzymes and signaling components; and (iii) inhibition of enzyme activity through temperature‐induced misfolding. While summarizing the current knowledge, we also highlight critical factors contributing to the deterioration of grain quality and propose potential strategies for enhancing the resilience of starch biosynthesis in wheat grain.

## Significance Statement

Combination of heat and drought stress reduces total yield and end use quality of grains by inhibiting starch biosynthesis through misfolding of enzymes, suppression of enzyme expression, and altering ABA and SnRK signalling pathways. This review discusses known bottlenecks in starch biosynthesis, highlight critical knowledge gaps, and proposes strategies for sustaining starch production in grains under harsh environmental conditions.

## INTRODUCTION

### Yield and grain quality under heat and drought

Cereals are a major source of macro‐ and micronutrients for both human and animal diets (Poutanen, 2012) of which wheat (*Triticum aestivum* L.) provides 18% and 19% of total dietary calories and protein, respectively (Erenstein et al., 2022). However, wheat production suffers from extreme weather events such as high temperatures and drought. For example, higher average temperatures, heat waves, and erratic precipitation patterns contribute to 20–49% of yield variation in wheat and other major crops, including maize, soy, and rice (Vogel et al., 2019). From 1981 to 2020, the probability of extreme temperatures in the wheat‐producing regions of China increased from 1 to 6%, and in the USA, from 1 to 17% (Coughlan de Perez et al., 2023). Significant yield losses of winter wheat in the United States Great Plains from 1982 to 2020 are attributed to compound hot–dry–windy events (HDW) with a 4% yield reduction per 10 hours of HDW during the period starting from heading until maturity (Zhao, Zhang, et al., 2022). Helman and Bonfil (2022) analyzed the effects of global warming, drought, and rising CO_2_ levels on yield in the top 12 wheat‐producing countries from 1961 to 2019. Their analysis revealed that a CO_2_ rise of ~98 μmol mol^−1^ increased the wheat grain yield by up to 7%, though warming of ~1.2°C and water depletion of ~29 mm m^−2^ reduced the yield by approximately 3–10%, depending on the region. Other studies project a 1.9% decrease in global wheat production due to climate change by 2050 (Pequeno et al., 2021), or 4.1–6.4% (Liu et al., 2016) and 6% (Zhao et al., 2017) for each 1°C of the average annual temperature increase (Box 1).

Box 1 **Summary of this review**

1. Heat and drought affect enzymatic activity in starch metabolism and transcription activity in starch biosynthesis.
2. Cross‐talk between ABA, T6P, and sucrose pathways balances yield and growth under heat and drought stresses.
3. The bottlenecks of the starch synthesis pathway can be targeted for developing a “Climate Smart” wheat.

**Figure 1:**
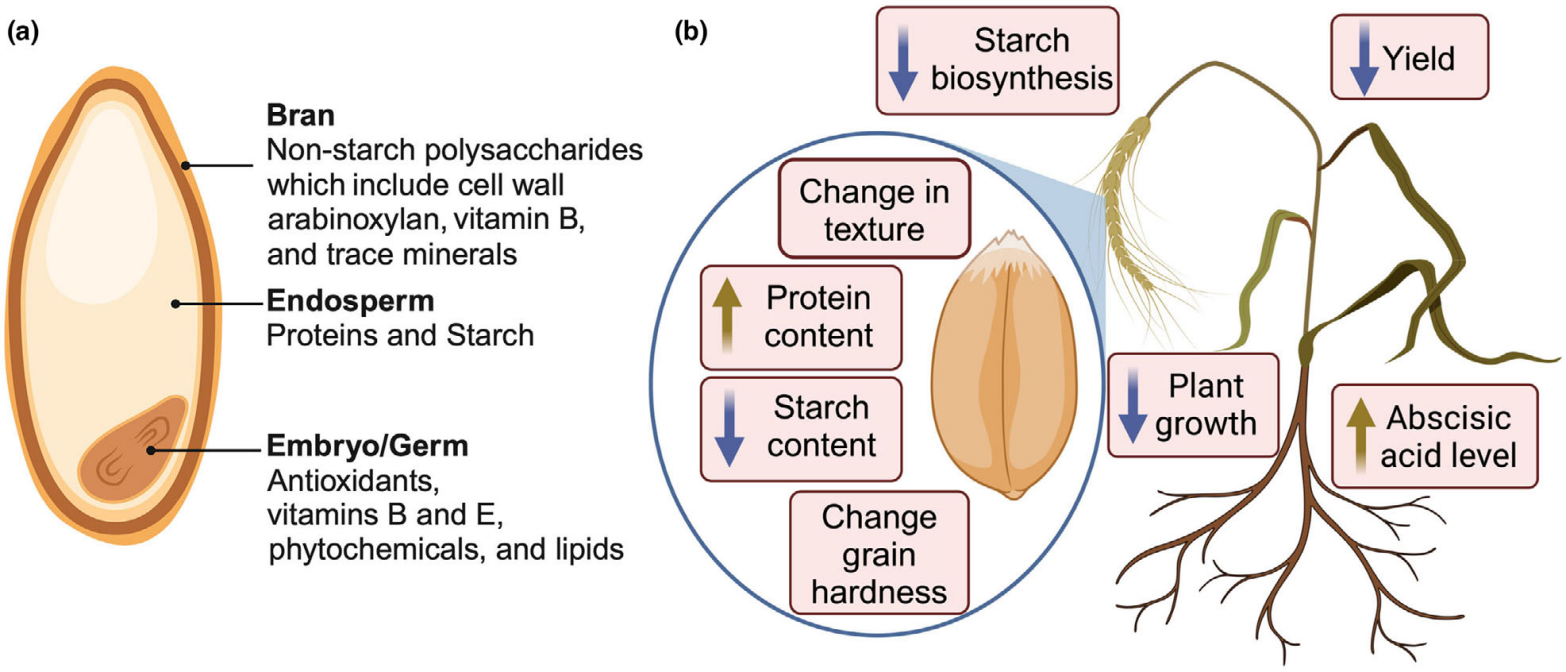
Heat and drought affect grain quality. (a) Nutrients in wheat grain. (b) Effects of heat and drought on wheat and grain quality. Created with BioRender.com.

The impact of higher ambient temperatures on biochemical and developmental processes causes a reduction of the nutritious value. The major nutrients in grains include carbohydrates (starch and other non‐starch polysaccharides), followed by proteins, lipids, and minerals (Figure 1a). The bulk of carbohydrates and proteins are stored in the endosperm, while the embryo contains antioxidants, vitamins B and E, and lipids. The outer grain layer, fiber‐rich bran, provides non‐starch polysaccharides including cell wall arabinoxylans, vitamin B, and trace minerals (Jayaprakash et al., 2022). Two types of starch molecules, amylose and amylopectin, make up between 60 and 75% of the seed's overall dry weight, whereas protein makes up 10–18% (Wieser et al., 2020). Storage gluten proteins represent around 80% of total grain proteins and can be grouped into aqueous alcohol‐soluble monomeric gliadins and insoluble polymeric gluten (Šramková et al., 2009). Moreover, the nutritional value of grains is determined by the content of essential amino acids (Wieser et al., 2020; Yang et al., 2022) of which leucine, phenylalanine, valine, and isoleucine are more abundant relative to lysine, methionine, tryptophan, threonine, and histidine (Khan et al., 2014; Siddiqi et al., 2020).

The content of most grain nutrients is affected by heat and drought (Mahdavi et al., 2022). The most critical are the reduction of grain starch content to 30–50% of total dry matter, increase of protein content (Table 1) (El Habti et al., 2020; Kumari et al., 2020; Mahdavi et al., 2022), and changes in grain hardness (Figure 1b) (Guzmán et al., 2022). Although the heat and drought also inhibit protein synthesis, the relative protein content of the total dry seed matter increases by 15–20% due to the decreased seed size (Daniel & Triboi, 2000; Mahdavi et al., 2022; Tanaka et al., 2021; Wan et al., 2022). Furthermore, the ratio of gliadin to glutenin increases relative to the grain from non‐stressed plants (Tanaka et al., 2021; Zhao, Tao, et al., 2022). The reduction of gluten content taken together with smaller seeds leads to a higher percentage of gliadin relative to total seed protein content whereas the percentage of glutenin decreases.

**Table 1:** Effects of heat and drought stresses on wheat grain quality

*Triticum aestivum* L. varieties	Duration of stress	Growth conditions	Starch content (tissue sample)	Protein content (tissue sample)	References
“Currawa,” “Koda,” “Mendos,” “Frame,” “Young,” “Gladius,” “Synthetic W7984,” “Odessa ES19565”	12 days post anthesis (3 days in abiotic stress)	Control: 22°C/15°C (day/night), Ψ^a^ = −0.3 mPa, Θg^b^ = 20% (g/g) Drought: Ψ^a^ = −0.6 mPa, Θg^b^ = 12% (g/g) Heat: 37°C/27°C (day/night)	Control: 0.52–0.56 g/g (grain DW) Drought: 0.46–0.52 g/g (grain DW) Heat + Drought: 0.45–0.51 g/g (grain DW)	‐	El Habti et al. (2020)
V‐19503, V‐19504, V‐19521, V‐19531, V‐19542, V‐19550, V‐19554, V‐19565, V‐19566, V‐19574, V‐19589, V‐19600, V‐19602, V‐19618	During the entire growth season (until maturity)	Control: Full irrigation Drought: Half irrigation	‐	Control: 12.5–14% (per grain) Drought stress: 14.8–16.1% (per grain)	Javed et al. (2022)
‘HD3059’ (heat tolerant) ‘BT‐Schomburgk’ (heat sensitive)	During grain filling (1 h of heat stress three times, for a total of 3 h)	Control: 26°C/22°C (day/night) Mild temperature stress: 32°C High‐temperature stress: 0°C	Control: 58–63% (endospermic tissue) Mild temp stress: 51–59% (endospermic tissue) High‐temp stress: 42–52% (endospermic tissue)	‐	Kumari et al. (2020)
“Xindong18,” “Xindong22”	Skipped irrigation at flowering and grain filling (until maturity)	Control: Full irrigation (1125 m^3^ hm^−2^ per irrigation) Drought stress: Skipped the last 2 irrigation cycles (2250 m^3^ hm^−2^ less)	Control: 63.5–67.5% (whole wheat flour) Drought stress: 60.5–65.5% (whole wheat flour)	‐	Li et al. (2023)
“Zhengmai 366”	10 days after anthesis until maturity	Control: 25°C/15°C (day/night) ~75% soil relative water content Heat stress: 32°C/22°C (day/night) Drought stress: ~50% soil relative water content Heat and drought stress: 32°C/22°C (day/night) ~50% soil relative water content	Control: 64–65% (per mg of grain) Heat stress: 41–42.5% (per mg of grain) Drought stress: 46–47.5% (per mg of grain) Heat and drought stress: 40–40.5% (per mg of grain)	‐	Lu, Hu, et al. (2019)
“CIMCOG 1‐60,” “Kouhdasht,” “Zagros,” “Karim,” “Dehdasht”	After anthesis and during grain development (termina heat stress)	Control: (2015–2016) 18 days over 32°C and 9 days over 35°C (2016–2017) 17 days over 32°C and 9 days over 35°C Heat stress: (2015–2016) 27 days over 32°C and 16 days over 35°C (2016–2017) 27 days over 32°C and 17 days over 35°C	Control: 63.48–72.63% (0.5 g of homogenized grain) Heat stress: 52.7–63.68% (0.5 g of homogenized grain)	Control: 11.08–12.60% (0.5 g of homogenized grain) Heat stress: 12.25–14.98% (0.5 g of homogenized grain)	Mahdavi et al. (2022)
“Alvand,” “Mihan,” “Rumor,” “Impression,” “Discus,” “Hybery”	During anthesis after anthesis normal irrigation continued for all treatments	Control: Irrigated twice during anthesis Mild drought stress: Irrigated once during anthesis Severe drought stress: No irrigation during anthesis	‐	Control: 12.5–16% (100 mg of whole flour) Mild drought stress: 13.5–16.5% (100 mg of whole flour) Severe drought stress: 14–17% (100 mg of whole flour)	Rekowski et al. (2021)
“Norin 61,” “Imam,” “Condor,” “Tagana,” “Bohaine,” “VYT11”	At heading (until maturity)	Control: 22°C/18°C (day/night) Heat stress: 38°C/18°C (day/night)	‐	Control: 11.5–13% (5 g of grain) Heat stress: 15.1–17.9% (5 g of grain)	Tanaka et al. (2021)
“Trisco”	10 days after anthesis (for 9 consecutive days)	Control: 21.7°C/14°C (day/night) Heat stress: 32°C/16°C (day/night)	‐	Control: 12–13% (1 g of whole flour meal) Heat stress: 13.5–14% (1 g of whole flour meal)	Zhang et al. (2019)
“Ningmai 13” (Weak gluten), “Zhenmai 12” (strong gluten)	Plants that were 7–9, 15–17, 23–25, and 31–33 days after anthesis were heat stressed 2 days for 4 times throughout the growing season	Control: 26°C/13°C (day/night) Heat stress: 35°C/22°C (day/night)	Control: 61.31–65.53% (10 g of whole meal) Heat stress: 56.84–57.51% (10 g of whole meal)	Control: 9.63–15.96% (whole wheat flour) Heat stress: 13.47–17.73% (whole wheat flour)	Zhao et al. (2022)

^a^ Soil water potential.

^b^ Gravimetric soil water content.

Decreased starch‐to‐protein ratios correlate with increased Zeleny sedimentation volume, loaf volume, and with slight increases being observed in other end‐use qualities such as ash content and water absorption (Balla et al., 2011; Lama et al., 2023; Mahdavi et al., 2022; Olckers et al., 2022; Satar et al., 2020). Furthermore, alongside higher ratios of gliadin to glutenin, heat and drought stress cause an increase in overall gluten content and quality (Guzmán et al., 2022). These changes in gluten composition influence end‐use quality through decreased dough elasticity and increased extensibility (Kłosok et al., 2021).

Wheat grains are primarily classified as either soft or hard textures and create either soft or hard flours (Shevkani et al., 2024). Soft flour contains lower protein levels and is fine and powdery, with minimal gluten, ideal for goods such as cakes, cookies, and pastries. Hard flours usually contain a higher protein content, which enables strong gluten formation and stretchiness/springiness, while being coarse and grainy, which is ideal for products such as pasta and bread (Paesani et al., 2021; Shevkani et al., 2024). Heat and drought stress generally result in harder grains, which are favorable for producing pasta and bread (Table 1).

### Knowledge gaps in understanding starch synthesis

Developing strategies for sustaining grain yield and quality under heat and drought stress relies on understanding stress responses in developing seeds at the molecular and physiological levels. One of the key pathways is the source‐sink relationship, broadly encompassing nutrient production in leaves, their translocation to developing seeds via the phloem, and packaging and storage in the endosperm and embryo. These processes are tightly regulated by a series of hormones and signaling cascades (Kumar et al., 2017; Yu et al., 2015) (Table 2). One such cascade is governed by the abscisic acid (ABA)‐mediated signaling pathway coupled with the sucrose non‐fermenting 1‐related kinases (SnRKs) pathway. SnRKs can be subdivided into three subfamilies: SnRK1/Snf1/AMPK (AMP‐activated protein kinase), SnRK2, and SnRK3 (Hrabak et al., 2003).

**Table 2:** Summary of effect of heat and drought stress on the starch biosynthesis pathway

Pathway	Generalized response to heat and drought stress		Key enzymes affected
	Source organs	Sink organs	
Synthesis of sucrose in source tissue	Decrease in sucrose biosynthesis	Sucrose not produced	Sucrose‐phosphate synthase/phosphatase
Sugar transporters and carriers in sink tissue	Increase in sucrose transporters	Varying responses	SUT SWEET
Sucrose synthase and invertase	Varying response	Increase in sucrose synthase and invertase	SuSy Invertase
Hexokinases and phosphoglucomutase	Varying responses	Hexokinase activity is stable or decreases, and phosphoglucomutase activity is stable or increases	Hexokinase Phosphoglucomutase
AGPase	Decrease in AGPase activity	Varying responses	AGPase
Starch synthesis and branching enzymes	A decrease in starch synthesis and branching enzymes	A decrease in starch synthesis and branching enzymes	Starch synthase Starch branching enzyme
Trehalose phosphate synthase and trehalose phosphate phosphatase	Increase in TPS and TPP activity	Varying response or increase of TPS, increase of TPP	TPS TPP
Abscisic acid	Increase in ABA	Increase in ABA	Zeaxanthin epoxidase 9‐cis‐epoxycarotenoid dioxygenase ABA aldehyde oxidase
SnRKs signaling	Activation of SnRK signaling	Activation of SnrRK signaling	SnRK bZIP

The ABA‐SnRK signaling network governs metabolic pathways including multiple reactions in both source and sink tissues responsible for starch biosynthesis. Under heat and drought stress, ABA‐SnRK causes accumulation of sucrose in grains (de Leonardis et al., 2015) that contributes to osmotic regulation and protection against heat‐induced damage at the expense of starch production. Engineering signaling events for sustaining sucrose flux into the starch biosynthesis pathway under heat and drought stress exemplifies how biochemical knowledge could contribute to sustaining food production. Another important consideration is that heat and drought inhibit starch synthesis by perturbing the activity of enzymes in the pathway (Table 2). Yet, our ability to mitigate these effects is hindered by limited mechanistic insights into how heat and drought stress affect biochemical reactions. Among the critical knowledge gaps remain the identification of bottlenecks in enzymatic activities, translating transcriptional changes of genes into the activity of corresponding chemical reactions, metabolic cross‐talk between different components of the starch biosynthesis pathway, and the impact of stress on sucrose transport.

**Figure 2:**
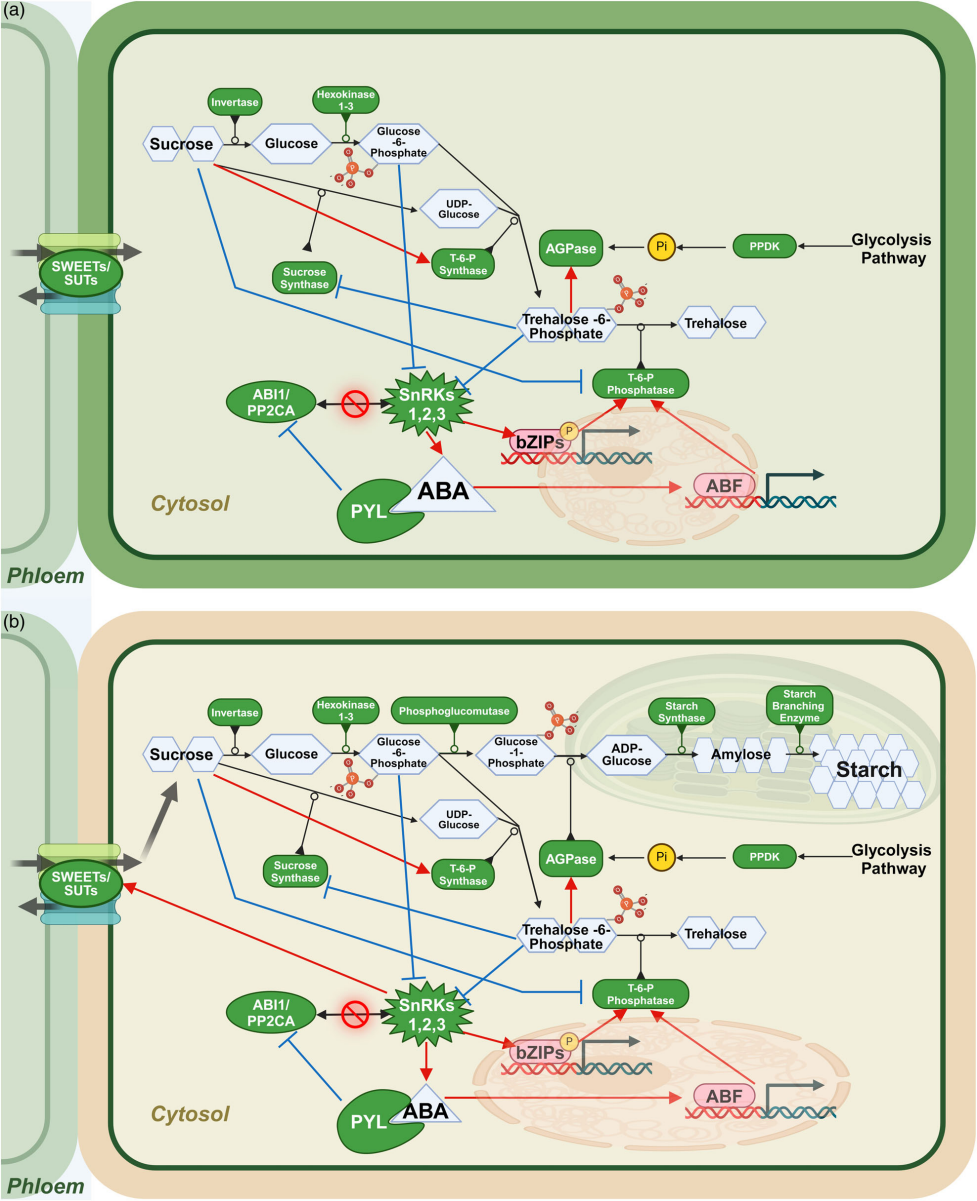
Schematic illustrating starch synthesis reactions in cereal source and sink tissue. (a) Sucrose is produced in source tissues and then transported by SWEETs/SUTs transporters into the phloem for delivery to sink tissues. Sucrose controls its production by inhibiting trehalose‐6‐phosphate (T‐6‐P) phosphatase, which inhibits sucrose synthase. Sucrose non‐fermenting‐1‐related protein kinases (SnRKs) play a key role by either directly or indirectly interacting with the ABA signaling pathway to activate transcription factors bZIPs or AREB/ABFs, respectively, for T‐6‐P phosphatase production. SnRKs influence the flux of sucrose into the sink tissues by regulating SWEETs/SUTs expression. (b) In the sink tissue, the sucrose gives rise to glucose‐6‐phosphate through the activity of invertase and hexokinase. Conversion of glucose‐6‐phosphate into glucose‐1‐phosphate by phosphoglucomutase initiates the starch synthesis pathway. Glucose‐1‐phosphate is transported into the amyloplast, where it becomes amylose via APGase. Starch is produced from amylose by the starch‐branching enzymes. Black arrows represent the general flow of the pathway. Red arrows represent potential upregulation, and blue lines show potential inhibition. Enzymes and proteins are highlighted in green, and metabolites are highlighted in blue. The red block icon shows that the PP2Cs are not interacting with SnRKs in the presence of ABA, rendering the SnRKs active. ABFs, ABA‐responsive elements binding factors; bZIP, basic leucine zipper family of transcription factors; PP2Cs, A type 2C protein phosphatases; PYL, Pyrabactin resistance 1 (PYR1)/PYR1‐like receptor protein; SnRKs, sucrose non‐fermenting 1‐related kinases; SUT, sugar transporter/sugar carrier; SWEET, sugars will eventually be exported transporter; TPP, trehalose‐6‐phosphate phosphatase; TPS, trehalose‐6‐phosphate synthase. Created with BioRender.com.

This review focuses on dissecting the interactions between stress signaling and metabolic networks responsible for starch biosynthesis in source and sink tissues. Starting with a general overview of the key processes under normal growth conditions, we follow with a holistic model of the effects of heat and drought on these pathways during the grain‐filling stage in bread wheat *T. aestivum* L. (Table 2, Figure 2). We discuss the potential of biochemistry for improving starch biosynthesis and what the remaining critical knowledge gaps are that hinder engineering wheat varieties with robust starch biosynthesis under heat and drought stress. Due to the limited number of studies on wheat seed development under stress conditions, we include relevant information generated in studies on grasses or other angiosperm species.

## RELOCATION OF SUGARS FROM THE SOURCE TO THE WHEAT GRAINS

### Synthesis of sucrose in source tissue

The synthesis and accumulation of starch in wheat grains begins with the biosynthesis of sucrose in photosynthetic (source) tissues (Figure 2a) (Emes et al., 2003; Nakamura, 2015) through the photosynthetic fixation of CO_2_. The fixed carbon enters the Calvin‐Benson cycle and becomes converted into triose phosphates in the chloroplast stroma (Sun et al., 2004). Triose phosphate is then transported to the cytosol and converted to fructose‐1,6‐phosphate by fructose‐1,6‐bisphosphatase. Then sucrose‐6‐phosphate synthase converts fructose‐1,6‐phosphate to sucrose‐6‐phosphate. Finally, sucrose‐6‐phosphate is converted into sucrose via the action of sucrose‐6‐phosphate phosphatase (Raven et al., 2004; Ruan, 2014). Sucrose can then be transported to the sink organs for the synthesis of starch (Figure 2b) (Hu et al., 2021; Kühn & Grof, 2010; Regmi et al., 2020).

### Remobilization of sugars via transporters/carriers

Sucrose can be transported for long distances via the symplastic pathway through the phloem (Turgeon, 2010). Anatomically, this pathway consists of sieve elements and companion cells within the vascular bundles (Braun et al., 2014). However, short‐distance transport of sucrose between adjacent cells via the apoplastic pathway is facilitated by transmembrane transporters. Two main types of transporters function in this pathway: sugar transporter/sugar carrier (SUT/SUC) and sugars will eventually be exported transporter (SWEET).

SUTs are a family of plant‐specific sucrose‐proton symporters, which are integral for the transport of sucrose where plasmodesmata are absent or not abundant (Hu et al., 2021). Uniquely, SUTs together with H^+^‐ATPase (SUT) can transport sucrose against the concentration gradient from the apoplast into the cytosol (Braun et al., 2014; Hu et al., 2021; Pegler et al., 2023). It has been shown that SUT expression in the aleurone of barley seeds contributes to the transport of sucrose into the endosperm (Pegler et al., 2023).

The symplastic pathway starts with sucrose loading into the phloem by SUT. The pivotal role of SUTs in seed development is supported by their expression pattern during developmental stages. For example, *TaSUT1* transcript levels were higher in source tissues, such as leaves, sheaths, and culms 4 days before heading and showed a significant decrease at 12 days after heading (Aoki et al., 2003). However, the transcription of *SUT* genes in the sink tissues increased during grain development (Aoki et al., 2003). Consistently, a knockout mutation of *OsSUT2* caused accumulation of sucrose in source leaves of rice due to a decrease in sucrose transport from the vacuolar to the cytoplasmic compartment (Eom et al., 2011).

SWEETs are a family of evolutionarily conserved channels that, unlike SUTs, transport sucrose passively across membranes along the concentration gradients (Pegler et al., 2023). Thus, SWEETs can transport sugars in or out of a cell without any energy input. Though SWEETs are not characterized in wheat, transcription of *SWEET* genes changes in response to heat and drought stress (Gautam et al., 2019). It has been shown that sucrose accumulated at higher levels in leaves of the maize *SWEET* mutant allele *zmsweet13a2b1c1* relative to the wild type (Bezrutczyk et al., 2018). Furthermore, transcription of *ZmSWEET13*s and *ZmSUT1* is higher in the bundle sheath cells compared to the mesophyll cells (Bezrutczyk et al., 2018). SWEETs play a critical role in seed development (Gautam et al., 2022). *SWEET* double knockout mutant in rice, *ossweet11:15*, caused an embryo abortion phenotype (Yang, Luo, et al., 2018). Mutants of *ZmSWEET4c* or *OsSWEET4* both showed defective grain filling (Sosso et al., 2015).

## STARCH BIOSYNTHESIS IN WHEAT GRAINS

### Sucrose hydrolysis by sucrose synthase and invertase

Upon reaching the sink tissues, sucrose undergoes irreversible hydrolysis by invertase, producing glucose and fructose (Figure 2b) (Ruan, 2014). Sucrose breakdown in the endosperm is catalyzed by sucrose synthase, resulting in fructose and UDP‐glucose (Chourey et al., 1998; Keeling et al., 1988; Tuncel & Okita, 2013). Invertases and sucrose synthases can be found in both soluble and insoluble forms in the pericarp and the endosperm. Three sucrose synthase genes (*TaSuSY1*, *TaSuSY2*, and *TaSuSY3*) are transcribed during the grain‐filling stage (Chevalier & Lingle, 1983; Hou et al., 2014; Volpicella et al., 2016). Furthermore, the activity of sucrose synthase and the abundance of *SuSY* transcripts in the wheat grain peak at the grain‐filling stage (Jiang et al., 2011). Mukherjee et al. (2015) detected all three *TaSuSY* transcripts in developing seeds between four and 8 days after anthesis and throughout the subsequent grain‐filling stages. The activity of sucrose synthase was elevated in the developing endosperms of wheat grains and correlated with the seed growth rate (Dale & Housley, 1986).

According to the intracellular localization, invertases form three groups: cell wall‐bound, cytoplasmic, and vacuolar. Each form can be characterized by solubility and optimal pH. The cell wall invertase is acidic, insoluble, and constitutes the main invertase activity (Sturm, 1999). It has been shown that cell wall invertase (CWINV) plays a role in both sucrose metabolism and development of wheat grain (Krishnan et al., 1985; Weber et al., 1997). Transcription of both *TaCWINV1* and *TaCWINV2* is upregulated during grain‐filling stages in wheat. The expression level declined at later developmental stages, and the *TaCWINV3* transcript could not be detected (Mukherjee et al., 2015). *CWINV* transcription peaked during early and late seed development stages in the pericarp of barley (Weschke et al., 2003), tomatoes (Shen et al., 2019), and maize (Chourey et al., 2006). Of the cytoplasmic and vacuolar invertases, which mainly function in root development and during biotic and abiotic stress, only vacuolar invertase was implicated in wheat grain development (Wang et al., 2022).

### Hexose sugar intermediates, hexokinases, and phosphoglucomutase

The products of sucrose hydrolysis, glucose, fructose, and UDP‐glucose are transported into the amyloplast, where glucose undergoes irreversible phosphorylation by hexokinase to produce glucose‐6‐phosphate. Hexokinases play a vital role in sugar sensing and are the only enzymes that can facilitate phosphorylation of glucose (Granot et al., 2013). There are two major types of hexokinases in plants: chloroplastic/plastidal and mitochondrial (Cho et al., 2006; Karve et al., 2008). Some monocots have a third type of hexokinase in the cytoplasm (Karve et al., 2010). Zhu et al. (2009) reported that higher hexokinase activity during the grain‐filling stage was accompanied by an increase in glucose‐6‐phosphate accumulation. Glucose‐6‐phosphate is then reversibly converted to glucose‐1‐phosphate by phosphoglucomutase. Phosphoglucomutase localizes in both the cytosol and the plastids during endosperm development in wheat (Esposito et al., 1999; Gu et al., 2021; Sangwan & Singh, 1987) and plays an important role in regulating the balance between the production of starch and the breakdown of carbohydrates (Figure 2b).

### ADP‐glucose pyrophosphorylase (AGPase)

Glucose‐1‐phosphate is converted to ADP‐glucose by ADP‐glucose pyrophosphorylase (AGPase). Plant AGPase consists of two small and two large subunits, with a total molecular weight ~200–240 kD (Saripalli & Gupta, 2015) of which the small subunits are evolutionarily more conserved. Two forms of AGPases are known in wheat: the heterotetramer of 51 and 54 kDa subunits forms in the source tissues, and the heterotetramer of 52 and 53 kDa subunits forms mostly in the cytosol of endosperm cells (Burton et al., 2002; Gómez‐Casati & Iglesias, 2002).

**Figure 3:**
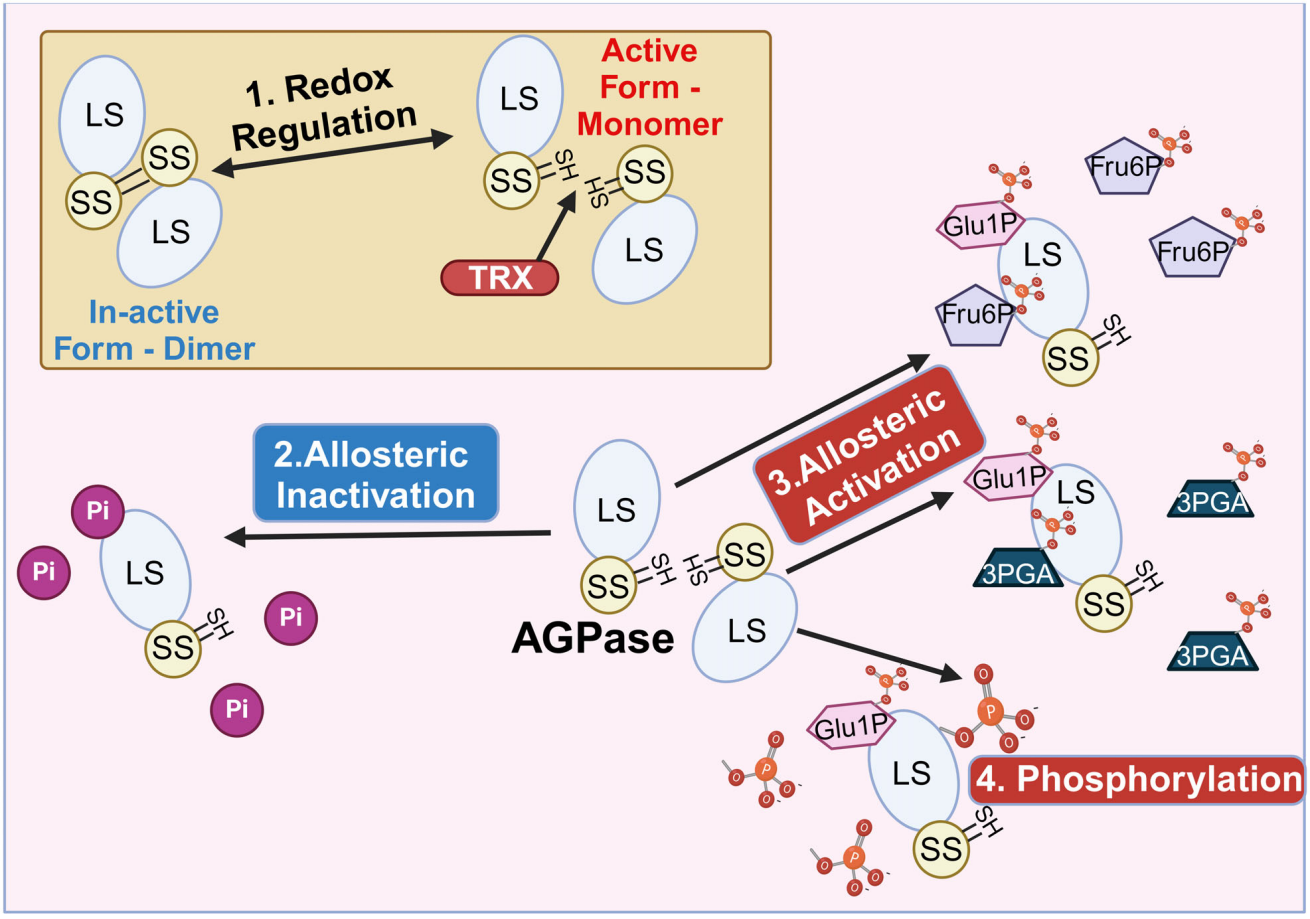
Regulation of AGPase activity. (1) In the dark, AGPase exists as an inactive heterotetramer formed by disulfide bonds between the small subunits. Under light conditions, thioredoxins activate AGPase by breaking the disulfide bonds. Active AGPase heterodimers can be allosterically activated through (2) increased affinity for substrates caused by high levels of 3PGA and Fru6P and (3) phosphorylation. (4) High levels of orthophosphate can inactivate AGPase activity. 3PGA, 3‐phosphoglyceric acid; Fru6P, fructose‐6‐Phosphate; Glu1P, glucose‐1‐phosphate; LS, large subunit; SS, small subunit. Figure created with BioRender.com.

AGPase activity in the source tissues is controlled through several distinct post‐translational mechanisms (Figure 3). (1) In the dark, AGPase exists as an inactive heterotetramer formed by reversible disulfide bridges between the two small subunits (Mugford et al., 2014). In the presence of light, however, electrons can be transferred from photosystem I to different thioredoxins (TRX) which then causes the formation of heterodimers and activation of AGPase (Figure 3, reaction 1) (Cejudo et al., 2019). (2) The allosteric activation of AGPase involves modification of the large subunit in the presence of high levels of 3‐phosphoglyceraldehyde, mediated by Pyruvate Phosphate Dikinase (Figure 3, reaction 2) (Prathap & Tyagi, 2020). The simultaneous activity of both redox and allosteric mechanisms leads to the highest AGPase activity (Mugford et al., 2014). Furthermore, there is evidence that AGPase in grasses becomes phosphorylated in the endosperm and that this phosphorylation correlates with a higher AGPase activity (Figure 3, reaction 3). SOS2 and CDPK1 kinases could be involved in this regulatory mechanism (Ferrero et al., 2018; Yu et al., 2023). Both the source organs and the endosperm forms of AGPase are inhibited by orthophosphate that acts on the large subunit (Figure 3, reaction 4).

### Starch synthesis and branching

Starch synthase uses ADP‐glucose to add a glucose unit to the non‐reducing end of an existing glucose polymer chain (Figure 2b). Granule‐bound starch synthase has two isoforms and facilitates the production of amylose, a linear α‐1,4 glucan, while soluble starch synthase has between 6 and 11 isoforms in cereals and mediates the elongation of amylopectin, a highly branched polymer of α‐glucose (Irshad et al., 2021; Jeon et al., 2010). Amylopectin is produced by the coordinated action of starch synthases, starch branching enzymes, and starch debranching enzymes. Starch branching enzymes cut segments from the chain of glucose and attach them elsewhere, creating a branch point. In contrast, starch debranching enzymes cleave off excessive or improperly placed branches, resulting in the formation of a tightly packed, semi‐crystalline amylopectin (Wu et al., 2013). The amylose and amylopectin molecules together form a concentric structure with amorphous and semi‐crystalline layers known as a starch granule. Amylose constitutes up to 25–30% of wheat grain starch, while the highly branched larger amylopectin comprises 70–75% of wheat grain starch (Kim & Kim, 2021). Starch granules provide an energy reserve and carbon source that can be utilized through hydrolysis by amylases down to glucose monomers when needed (Tetlow, 2011; Thitisaksakul et al., 2012).

### Trehalose‐6‐phosphate, a sucrose mirror

Glucose‐6‐phosphate and UDP‐glucose can also be used as substrates for trehalose‐6‐phosphate production in a reaction catalyzed by the enzyme trehalose‐6‐phosphate synthase (TPS). Subsequently, trehalose‐6‐phosphate phosphatase (TPP) facilitates the conversion of trehalose‐6‐phosphate to trehalose (Paul et al., 2020). Trehalose‐6‐phosphate and trehalose are ubiquitous among plants, green algae, and even prokaryotes (Lunn et al., 2014). In wheat, trehalose‐6‐phosphate levels in the grain reach 79 nmol g^−1^ of fresh weight between 1 and 10 days after anthesis, which closely mirrors sucrose levels in the grain (70 nmol g^−1^ of fresh weight; Martínez‐Barajas et al., 2011). This highlights that trehalose‐6‐phosphate levels follow similar patterns as sucrose, whereby high levels of trehalose‐6‐phosphate are accompanied by high levels of sucrose and vice versa, suggesting that trehalose‐6‐phosphate also functions as a signal molecule of carbon status and availability (Choudhary et al., 2022).

## SIGNALING CASCADES FOR THE REGULATION OF STARCH BIOSYNTHESIS

### Abscisic acid, a universal hormone

**Figure 4:**
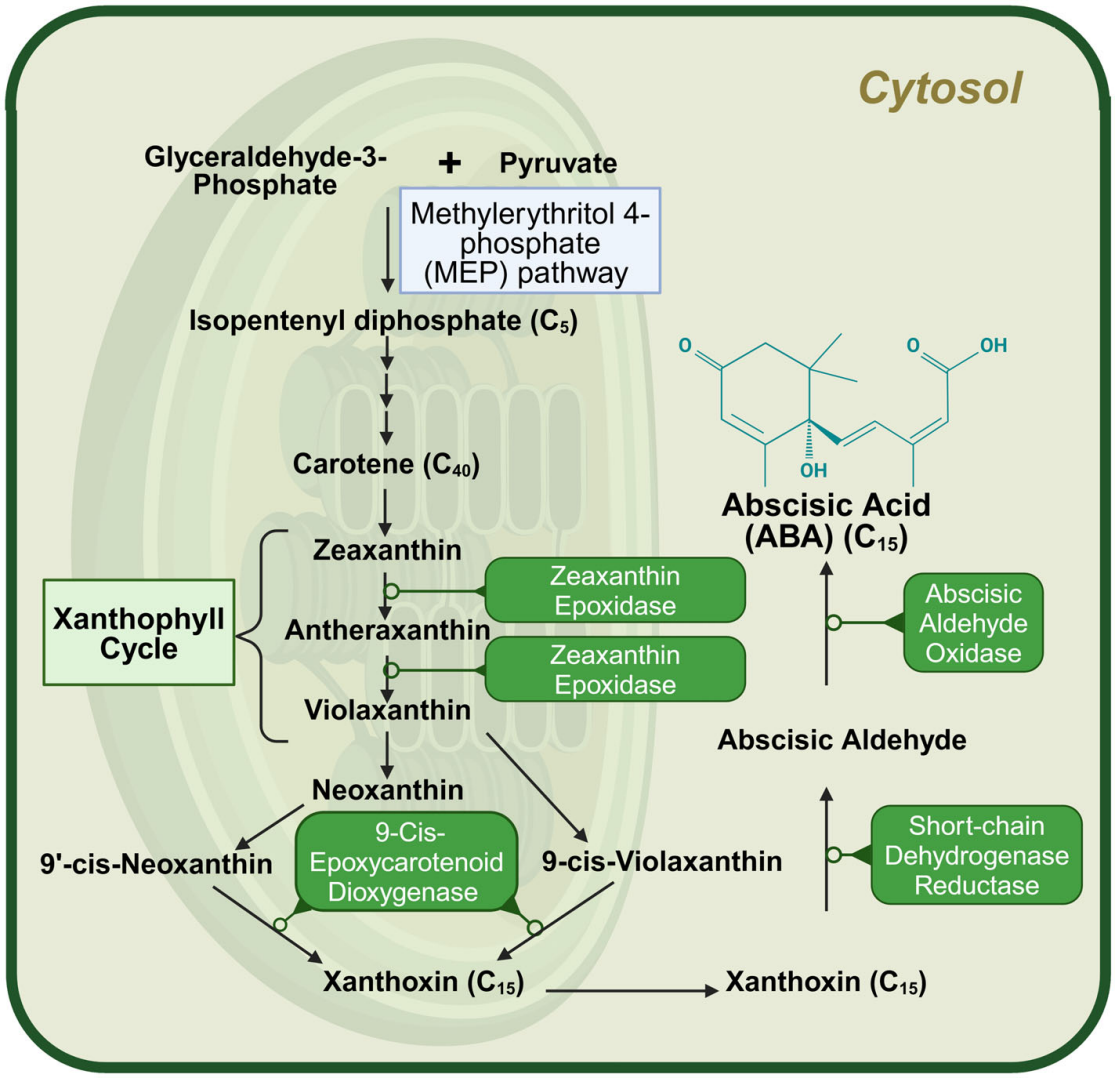
Abscisic acid (ABA) biosynthetic pathway in plants. ABA is synthesized *de novo* in the plastids from a C40 epoxy carotenoid precursor. The initial intermediates originating from glycolysis products, pyruvate, and glyceraldehyde‐3‐phosphate to isopentenyl diphosphate via the Methyl Erythritol Phosphate pathway are not depicted here. The C15 intermediate xanthoxin is converted into ABA through a two‐step reaction involving ABA‐aldehyde in the cytosol. The enzymes involved in ABA biosynthesis are highlighted in green ovals. Created with BioRender.com.

Although frequently referred to as a “stress hormone,” ABA, in addition to responding to a range of abiotic stresses, plays a key role in plant growth and development. ABA biosynthesis is well characterized in Arabidopsis and appears to be conserved across angiosperms (Xiong & Zhu, 2003). The carotenoid pathway involving the C40 epoxy carotenoid acts as a precursor for de novo ABA synthesis (Finkelstein, 2013). The ABA biosynthetic pathway then involves the epoxidation of zeaxanthin and antheraxanthin to violaxanthin in plastids via zeaxanthin epoxidase, also known as the xanthophyll cycle (Kress & Jahns, 2017). The second function of the pathway is the non‐photochemical quenching of excess light energy in PSII. The first committed step in ABA synthesis involves the oxidative cleavage of 9‐cis‐neoxanthin by the 9‐cis‐epoxycarotenoid dioxygenase, yielding a C15 intermediate, xanthoxin. Xanthoxin is further converted to ABA‐aldehyde by a short‐chain alcohol dehydrogenase/reductase. Finally, ABA aldehyde oxidase catalyzes the last step of ABA synthesis, converting ABA‐aldehyde to ABA (Finkelstein, 2013) (Figure 4). Zeaxanthin epoxidase, 9‐cis‐epoxycarotenoid dioxygenase, and ABA aldehyde oxidase are all upregulated by stress (Tuteja, 2007), whereas short‐chain alcohol dehydrogenase/reductase is upregulated by sugars (Cheng et al., 2002).

Given that the ABA biosynthetic enzymes are localized in chloroplasts, a significant amount of ABA synthesis occurs in mature leaves, specifically in the phloem companion cells and the stomata guard cells (McAdam & Brodribb, 2018). However, ABA synthesis still occurs in different plant tissues, including roots, and is transported through vascular bundles in both directions between the roots and shoots (Finkelstein, 2013). This fact underscores non‐linear relationships between ABA signaling under heat and drought stress in specific tissues and the regulation of grain development.

### Abscisic acid contributes to seed development

ABA governs the grain development and nutrient accumulation on two scales: the whole plant and the seed (Govind et al., 2011; Seiler et al., 2011). On the whole‐plant scale, ABA induces leaf senescence, which in turn enables reallocation of nutrients, particularly sugars and amino acids, from source to sink organs (Zhao et al., 2017). On the seed scale, ABA abundance forms two peaks: (i) during early developmental stages, most likely by ABA produced in the maternal tissues; and (ii) during later developmental stages by ABA produced in the embryo (Xiong & Zhu, 2003).

Seiler et al., 2011 observed high transcript levels of genes encoding enzymes responsible for initial ABA synthesis in barley seeds, including zeaxanthin epoxidase *1*, 9‐cis‐epoxycarotenoid dioxygenase *2*, and short‐chain alcohol dehydrogenase/reductase *1*. This fact supports the ability of embryos to synthesize ABA *de novo* (Figure 4). Interestingly, ABA aldehyde oxidase and molybdenum cofactor sulfurase required for ABA aldehyde oxidase activity in catalyzing the last step of ABA biosynthesis, were not found in wheat grains during the early stages of seed development. Plausibly, precursors of ABA are synthesized during the initial stages of seed development and then utilized for de novo ABA synthesis in later stages. During the second peak, around 16 days after fertilization, *ABA aldehyde oxidase 1* transcripts were ~5000‐fold more abundant in seeds in comparison with flag leaves (Seiler et al., 2011). This evidence supports the hypothesis that the second ABA peak results from de novo ABA synthesis.

The exact mechanism by which ABA influences enzymes involved in starch biosynthesis in developing seeds remains poorly understood. ABA‐mediated signaling depends on ABA‐insensitive (ABI) transcription factors, specifically ABI5 in seeds and ABA‐responsive elements (ABRE) binding factors (AREB/ABFs) in vegetative tissues. Many of these transcription factors belong to bZIP (basic leucine zipper) families that bind to ABRE and regulate the expression of downstream genes (Skubacz et al., 2016). As was shown in Arabidopsis, ABA can control gene expression through the synergistic interaction of ABI4 with ABI5 and other bZIP transcription factors (Govind et al., 2011).

### Sucrose non‐fermenting‐1‐related protein kinases (SnRKs) and their downstream bZIP targets

SnRKs play diverse roles in regulating plant metabolism. Based on the sequence similarity, SnRK1/Snf1/AMPK is evolutionarily related to the yeast *sucrose non‐fermenting‐1* gene and acts as an energy sensor. The other two members of the family, SnRK2 and SnRK3, the products of gene duplication events, specifically interact with the components of ABA and stress signaling pathways (Halford & Hey, 2009). SnRKs regulate development and stress responses by phosphorylating and activating members of the bZIP family of transcription factors (Jakoby et al., 2002). For instance, in *Arabidopsis thaliana*, SnRK1 activates bZIP1, 2, 11, 53, and 63, which modulate metabolic processes associated with energy reallocation (Mair et al., 2015; Matiolli et al., 2011). Additionally, the wheat SnRK2 homolog, PKABA1, becomes upregulated together with the AREB/ABFs gene *TaABF* to govern grain maturation and dormancy by suppressing gibberellic acid‐induced gene expression (Johnson et al., 2002).

The direct relationship between SnRKs and wheat grain quality remains enigmatic. One mechanism could involve activation of bZIPs that control starch and protein content, thereby influencing seed quality in cereals (Kumar et al., 2018). For instance, overexpression of *TabZIP28* in wheat seeds enhances starch synthesis by activating the transcription of cytosolic AGPase (Song et al., 2020). Notably, BLZ1/OHP1/bZIP63, a wheat endosperm transcription factor with a role in seed development, contains the SnRK1 recognition site and could be regulated by the SnRKs‐bZIP signaling pathway (Curtis et al., 2019).

### Cross talk between SnRKs and trehalose‐6‐phosphate signaling

SnRKs cooperate with the trehalose‐6‐phosphate signaling pathway in regulating starch accumulation in grains. It has been shown that trehalose‐6‐phosphate regulates both SnRKs and AGPase: the reduction of trehalose‐6‐phosphate abundance in developing wheat seeds is accompanied by lower SnRK activity (Liu, Si, et al., 2023) and the diminished trehalose‐6‐phosphate abundance in Arabidopsis leaves causes lower reductive activation of AGPase. Conversely, Arabidopsis leaves with higher trehalose‐6‐phosphate levels displayed an elevated reductive activation of AGPase (Kolbe et al., 2005). How trehalose‐6‐phosphate contributes to post‐translational regulation of the enzymatic activities remains unclear. Additionally, a direct interaction between AREB/ABFs and TPP was shown in Arabidopsis, linking ABA to trehalose‐6‐phosphate signaling (Lin et al., 2020). The SnRK‐ABA‐trehalose‐6‐phosphate signaling nexus plays a critical role in regulating starch synthesis under stress conditions.

## WHOLE‐PLANT RESPONSES TO HEAT AND DROUGHT STRESS

### The ABA‐AREB/ABFs‐SUT/SWEETs pathway contributes to sucrose reallocation during stresses

ABA signaling through SnRK induces nutrient remobilization from source to sink tissues by promoting leaf senescence and by increasing sucrose transport (Ghate et al., 2019; Han et al., 2017; Pattanagul, 2011; Yang et al., 2014). One crucial factor in promoting sucrose transport is the higher rate of phloem unloading in the sink organs by ABA‐induced up‐regulation of sucrose transporters (Peng et al., 2003). Ghate et al. (2019) reported that drought stress in sorghum causes a higher transcription level of several *AREB/ABF* genes, but also genes involved in sucrose transport including *invertase*, *SUTs*, and *SWEETs*. The promoters of all these genes contain the ABRE motif.

Comparison of heat‐tolerant and heat‐sensitive wheat genotypes revealed five differentially expressed *SWEET* genes (Gautam et al., 2019). *TaSWEET14h‐1B* and *TaSWEET2b2‐3A* were downregulated (−1.5 to −2.5 relative fold change) in the susceptible compared to the tolerant line, whereas TaSWEET*15a‐7D*, *TaSWEET17c‐5A*, and *TaSWEET2a1‐6B* were upregulated (1 to 5.5‐fold) in susceptible relative to the tolerant genotypes. Furthermore, heat stress in Arabidopsis increased carbon export from the source tissue and up‐regulated transcription of *AtSWEET11* and *AtSWEET12* (Durand et al., 2016). Gautam et al. (2019) showed that drought stress promoted transcription of *TaSWEET2b2‐3A* and *TaSWEET16a‐4A* by 9‐ to 12‐fold, respectively, in a drought‐sensitive genotype after 1 or 6 h of the treatment (Gautam et al., 2019). This evidence suggests that SWEETs ameliorate the adverse effect of heat and drought stresses on sink tissues by increasing the reallocation of sugar from the source tissues.

SUTs become essential in reallocating sugar from the sieve elements and companion cell complexes to their surrounding cells under heat or drought due to the low number of plasmodesmata between these cells (Hu et al., 2021). Expression of wheat SUTs depends on the genotype, growth stage, and environmental conditions. Al‐Sheikh Ahmed et al. (2018) showed up‐regulation of *SUT1* in grain and stem tissue in response to drought stress in a susceptible wheat variety and down‐regulation of *SUT1* in a drought‐tolerant variety. Transcription of *SUT* genes was sensitive to heat stress (2 h at 42°C) in a heat‐susceptible wheat variety. An *SUT* encoded by *CL3697Contig1* was the most down‐regulated transcript under heat stress with a log2‐fold change of −10.64 (Kumar et al., 2015). Comparing the impact of heat stress on genes encoding enzymes associated with sucrose metabolism in chickpea, *Cicer arietinum*, and wheat showed down‐regulation of *SUT* genes in both species (Kaushal et al., 2013). In conclusion, the reduction of *SUT* transcription would likely cause a lower sucrose availability and a lower rate of starch biosynthesis in the sink organs.

### ABA interaction with the starch synthesis pathway during heat and drought stresses

In addition to the above mechanisms, ABA governs starch synthesis in grains by controlling the transcription of key enzymes in this pathway. For example, ABA promoted the transcription of barley sucrose synthase *SUS1* (*HvSUS1*), the AGPase small subunit (*HvAGP‐S1*), and β‐amylase (*HvBAM1*; Govind et al., 2011). The accumulation of ABA in grains correlated with higher activity of the starch biosynthetic enzymes including sucrose synthase, soluble starch synthase, starch branching enzyme, and AGPase (Yang et al., 2004). However, how ABA activates gene transcription and the enzymatic activity of the starch biosynthesis pathway remains poorly understood.

### SnRK‐bZIPs pathway in stress responses

As discussed above, ABA promotes activity of SnRKs by inhibiting PP2C (Kumar et al., 2019). In addition to this mechanism, there is transcriptional activation of SnRK genes in response to stress, for example, drought promoted transcription of *TaSnRK2.4‐B*, *TaSnRK2.7A*, *TaSnRKK3.35*, and *TaSnRK3.37* in wheat leaf (Jiang et al., 2022). However, our knowledge about the expression of *SnRKs* under abiotic stresses in wheat seeds remains limited. Transcriptomic analysis during the grain‐filling stage revealed a 7.7‐fold increase of *SnRK1* transcript in heat‐sensitive wheat lines compared to the heat‐tolerant lines when 38°C/20°C (day/night) heat stress was applied for 3 days at the late‐grain filling stage (27 days post‐anthesis) (Rangan et al., 2020).

The SnRKs‐bZIP pathway links sugar signaling in regulating plant development with responses to heat and drought stress by controlling transcription of stress resilience genes (Liu et al., 2013). Numerous studies showed transcriptional up‐regulation of several *TabZIP* genes in both source and sink tissues of wheat under heat stress (Agarwal et al., 2019) (Figure 2). Overexpression of wheat *TabZIP* (Traes_7AL_25850F96F.1) in Arabidopsis enhanced seed setting under drought stress. Furthermore, other members of the gene family, *TabZIP60* (Zhang et al., 2015), *TaABP1* (Cao et al., 2012), and *TabZIP8* (Wang et al., 2021) can improve drought resilience. There is currently no information about the downstream targets of wheat bZIPs and how they control stress tolerance.

There is evidence that a similar pathway functions in other crops. Overexpression of *OsbZIP71* in rice increases the seed‐setting rate under drought from 38% in the wild‐type control to 58–63% in the transgenic line (Liu et al., 2014). OsbZIP62 interacts with SnRK2 and enhances plant tolerance to drought and oxidative stress by upregulating the wax synthesis protein (OsGL1, *LOC_Os02g56920*) that contributes to increasing wax thickness in leaf cuticle (Yang et al., 2019). Furthermore, OsbZIP66 (Yoon et al., 2017), OsbZIP62 (Lee & Song, 2023), OsbZIP23, OsbZIP45 (Park et al., 2015), OsbZIP12 (Joo et al., 2014), OsbZIP16 (Pandey et al., 2018), OsbZIP42 (Joo et al., 2019), and OsbZIP46 (Chang et al., 2017) are activated by SnRKs, thereby improving resistance to drought, heat, and cold stresses.

## EFFECT OF ABIOTIC STRESS ON STARCH METABOLISM ENZYMES IN WHEAT

### Thermal kinetic window (TKW) of enzymes

Heat stress can affect enzymatic activity by causing protein unfolding. Every enzyme can be characterized by a thermal kinetic window (TKW), defined as the optimal temperature range at which the Michaelis constant is equal to or below 200% of the minimum observed value (Burke et al., 1988). An alternative definition of the TKW is the range of temperature for optimal enzymatic function (Burke et al., 1988; Fitter & Hay, 2012). The TKW depends on the enzyme and the species. For example, the TKW of glyoxylate reductase for NADH during conversion of UDP‐glc to UDP‐GlcA is between 17.5 and 23°C in wheat (*T. aestivum* L.) and 23.5 and 32°C in cotton (*Gossypium hirustum* L.; Burke et al., 1988; Litterer et al., 2006).

The conformation of enzymes and their activity can be sustained by chaperones. Hu et al. (2018) investigated the role of the heat shock factor *TaHsfC2a*, which is highly expressed in wheat grains during grain filling. Overexpression of *TaHsfC2a* (70‐fold higher than wild‐type) resulted in the upregulation of a group of heat protection genes including *TaHSP70d* (~2‐fold increase). In wild‐type plants, transcription of *TaHsfC2a* in leaves was up‐regulated by ~1.4‐fold in response to drought or ABA treatment and was not affected by heat stress. However, overexpression of *TaHsfC2a* contributed only to thermotolerance but not drought tolerance, suggesting the existence of a heat protection mechanism in developing wheat grains via the ABA‐dependent expression of chaperones (Hu et al., 2018). This mechanism appears to be conserved across plant species as ABA could induce expression of heat shock proteins during grain‐filling stages under high‐temperature stress in maize (Tao et al., 2016).

### Heat and drought starch metabolism

Enzymes responsible for the hydrolysis of sucrose in the sink tissues are sensitive to heat and drought stress. The expression pattern of genes encoding several key enzymes showed that most were downregulated in both heat and drought stress, which caused a major change in the enzyme activity, ultimately reducing starch synthesis (Lu, Hu, et al. 2019). The enzymatic activity of both invertase and sucrose synthase in the developing wheat grain decreased during heat stress (5–9°C above the normal temperature of 28.7°C) from 6.3 ± 0.27 nmol min^−1^ mg protein^−1^ to 5.4 ± 0.09 nmol min^−1^ mg protein^−1^ and from 25.7 ± 0.5 nmol min^−1^ mg protein^−1^ to 20.1 ± 0.7 nmol min^−1^ mg protein^−1^, respectively (Bansal et al., 2013). Concomitantly with the reduction of enzymatic activity, the content of glucose in the developing grain was reduced by 14 to 17.7% under stress conditions in both tolerant and sensitive genotypes relative to the normal growth conditions, while glucose‐6‐phosphate content was also lower by 15.6 to 19.2% (Bansal et al., 2013) compared to the normal growth conditions.

Another phenotype of heat stress was a lower transcription level of genes encoding cell‐wall invertases (log_2_‐fold change of −0.6 to −2.1; Qin et al., 2008). The activity of soluble invertase was more sensitive to drought stress than the insoluble invertase in the wheat grains and leaves. Also, the concentrations of glucose, fructose, and sucrose in the flag leaves were reduced by 55.7, 46.9, and 37%, respectively, under drought stress (Saeedipour & Moradi, 2011). Activity of both sucrose synthase and invertase was lower under drought stress in wheat (Kutlu et al., 2021). Drought stress caused lower activity of vacuolar and cell wall‐bound acid invertase during grain development in maize and reduction of hexose sugars (Zinselmeier et al., 1995). Enzymatic activities and gene transcription of both sucrose synthase and invertase were upregulated in leaves of soybeans in response to drought (Du et al., 2020). Although the sugar content, enzymes, and their expression levels increased in developing seeds under short periods of drought, prolonged exposure to drought caused a reduction of these parameters (Du et al., 2020).

### TPS and TPP sense heat and drought stress

Wheat and other monocots exhibit variable changes in *TPS* and *TPP* gene expression in response to heat and drought (Luo et al., 2022; Yu et al., 2020). The wheat genome contains 25 *TPS* genes, of which 13 are transcriptionally downregulated by heat stress and 3 *TPS* genes are upregulated by up to 2‐fold (Paul et al., 2020; Tomás et al., 2022). The *TPP* gene family in wheat comprises 31 members. Transcriptional analysis showed that drought stress causes upregulation of 3 *TPPs* in leaf (*TPP3*, *TPP7*, *TPP10*) and 4 *TPPs* (*TPP5*, *TPP6*, *TPP7*, *TPP11*) in grain by up to 5‐fold (Du et al., 2022; Paul et al., 2020). These changes are accompanied by the reduction of trehalose‐6‐phosphate levels. Reduced trehalose‐6‐phosphate levels result in lower activity of AGPase (Kolbe et al., 2005) and higher activity of SnRK (Liu, Si, et al., 2023). Explaining the complexity of the above transcriptional responses remains challenging due to a lack of data about the enzyme kinetics of plant TPS and TPP under heat stress, with the data being limited to thermophilic prokaryotes (Cross et al., 2017; Pan et al., 2002).

### AGPase activity is sensitive to heat and drought stresses

Heat stress causes lower activity of key enzymes involved in starch biosynthesis, such as AGPase, starch synthase, granule‐bound starch synthase, and starch branching enzymes (Lal et al., 2022; Ribeiro et al., 2022). Heat stress could affect the activity of AGPase at the transcriptional level. Lu et al. (2019) reported that 32/22°C (day/night) conditions applied for 10 days after anthesis resulted in downregulation of *AGPase* isoforms (*AGPS1‐a*, *AGPS1‐b*, *AGPS2*, *AGPL1*, and *AGPL2*), and lower availability of substrate for starch biosynthesis in wheat seeds. Amylose, amylopectin, and total starch content were reduced by 10.2, 13.4, and 12.7%, respectively. Mild drought conditions defined as 50% of the relative soil water content, caused a reduction of amylose, amylopectin, and total starch content in grains by 4.2, 6, and 5.6%, respectively, whereas the combination of heat and drought led to a reduction of these components by 14.9, 18.7, and 17.8%. Furthermore, genes encoding enzymes responsible for ADP‐glucose conversion to amylopectin, including synthases, branching and debranching enzymes, and phosphorylases were also downregulated (Lu, Hu, et al. 2019).

Exposure to heat stress caused reduction of AGPase, soluble starch synthase, and starch branching enzyme activities in maize grain by 21.3–43.1, 19.1–29.2, and 7.0–45.6%, respectively, in the first year of studies and by 1.8–78.5, 21.4–51.2, and 11.0–48.0% in the following year (Yang, Gu, et al., 2018). The elevated activity of AGPase in developing rice grains was associated with increased transcription level of the ADP glucose pyrophosphorylase large subunit 3 in a drought‐tolerant genotype, whereas in a drought‐sensitive genotype transcription of ADP glucose pyrophosphorylase small subunit 2 and ADP glucose pyrophosphorylase large subunit 3 were upregulated. Higher starch synthase activity in developing grains was associated with the increased expression of soluble starch synthase variants (*SSIIB*, *SSIVA*, and *SSIVB*) in a drought‐tolerant genotype and *SSIVB* in a drought‐sensitive genotype (Prathap & Tyagi, 2020).

## APPLICATION OF BIOCHEMICAL WISDOM TO BREEDING PRACTICES

The most logical way to harness the power of small signaling molecules ABA and trehalose‐6‐phosphate is through their exogenous application. It has been shown that the application of ABA enhances grain filling under stress by regulating starch biosynthesis, antioxidant defense, and hormone balance in wheat (Lamlom et al., 2025; Yang et al., 2013; Zulfiqar et al., 2024), rice (Liu, Zhong, et al., 2023), and maize (Yu et al., 2024). One study reported the reduction of grain weight and water use efficiency by exogenous ABA application under severe drought conditions (Luo, Li, et al., 2021). Detailed analysis revealed that ABA concentrations up to 200 ppm benefit drought tolerance (Ahmadi & Baker, 1999; Zulfiqar et al., 2024). Thus, the efficacy of ABA sprays depends on optimal dosage and severity of drought stress as well as the developmental stage.

Although there is currently no data on the exogenous application of trehalose‐6‐phosphate, it was shown that the application of trehalose protects PSII integrity in wheat under heat stress (Luo, Liu, et al., 2021) and improves carbon assimilation in maize and wheat (Zhang et al., 2022). Under drought stress, the application of trehalose causes higher antioxidant capacity in wheat and corn and protects photosystem II integrity in corn (Han et al., 2024). Despite exogenous applications can help mitigate heat and drought stress in trials, their practical significance in dryland farming is limited by the application costs. A more cost‐efficient approach for exploiting these pathways could be through metabolic engineering. Several studies have already successfully explored genetic tools for engineering ABA, trehalose‐6‐phosphate, and other pathways for advancing resiliency and seed quality under stress (Table 3).

**Table 3:** List of genes that have been utilized to enhance small grains traits under stress

Pathway	Genes engineered	Technology	Outcome	References
AGPase	*Sh2r6hs* (an altered AGPase large subunit)	Ectopic expression of a maize gene in wheat	38% seed weight per plant and 31% plant biomass increases	Meyer et al. (2004)
	Bt2 and *Sh2* (two subunits of AGPase)	Overexpression in maize	9% starch content and 15% grain weight increases	Li et al. (2011)
SnRK	*OsNAC23*	Overexpression in rice	Increased yields by 13–17%	Li et al. (2022)
	*TaSnRK 2.9‐5A*	QTL marker breeding in wheat	Yield contributing trait	Ur Rehman et al. (2019)
TPS/TPP	*TaTPP‐7A*	Overexpression and RNAi in wheat	Increased in starch synthesis gene expression and higher sucrose and trehalose content	Liu et al. (2023)
	*TaTPP‐6AL1*	QTL marker breeding in wheat	Increase in wheat species that contain the 6AL1a allele as opposed to the 6AL1b allele	Zhang et al. (2017)
ABA	*TabZIP28*	Overexpression in wheat	Enhanced starch synthesis	Song et al. (2020)
	*OsbZIP71*	Overexpression in rice	Higher yield under drought	Liu et al. (2014)
Sucrose hydrolysis	*OsSus3*	QTL marker breeding in rice	Higher heat tolerance during the ripening stage	Takehara et al. (2018)
Glycogen branching enzyme	*OsglgC*	Ectopic expression of an engineered *E. coli* gene in rice	11% seed weight increased	Dauvillée et al. (2005)
Heat shock protein	*HSP70*	Ectopic expression of *Physcomitrium patens HSP70* in rice	Higher proline content, increased hydroxyl radical scavenging, lower hydrogen peroxide content, and seedling survival under heat stress	Kou et al. (2023)

Attempts to engineer the trehalose‐6‐phosphate metabolic pathway have thus far generated encouraging results. Overexpression of the *TaTPP‐7A* gene in wheat caused upregulation of starch biosynthesis enzymes, a lower ratio of amylose to amylopectin, and increases in the content of sucrose, trehalose, and soluble sugars but did not affect overall grain starch content under normal growth conditions (Liu et al., 2023). Zhang et al. (2017) created a molecular breeding marker on the gene *TaTPP‐6AL1* associated with enhanced grain weight in wheat. Identification of a heat‐responsive *TPS* isotype, *TPS5*, in wheat offers an alternative approach for engineering trehalose‐6‐phosphate signaling under stress (Xie et al., 2015). A molecular marker in a wheat *SnRK* gene contributes to yield and can be used in breeding programs (Ur Rehman et al., 2019).

Kou et al. (2023) could improve heat and drought tolerance in rice by overexpressing the *HSP70* gene from *Physcomitrium patens*. Hence, increasing the expression of heat shock proteins could enhance heat and drought stress tolerance. Implementing this potentially promising approach in breeding programs requires more research on how genes encoding heterologous or endogenous heat shock proteins affect yield and end‐user traits. Promoters of heat shock proteins enable the activation of resistance pathways under stress conditions. For example, the promoter of the Arabidopsis *HSP18.2* gene, *HSE‐COR15A*, was used for heat‐inducible gene expression (Yoshida et al., 1995). Recently, a heat shock–inducible CRISPR‐Cas9 gene‐editing system was developed based on the *HSE‐COR15A* promoter to knock down gene expression in Arabidopsis under heat shock (Liang et al., 2023). Similar strategies can be devised for modulating the activities of signaling pathways in response to heat stress in wheat.

## CONCLUDING REMARKS AND OUTSTANDING QUESTIONS

Engineering “climate‐smart” varieties with more stable yield and balanced protein‐starch ratio in grain under abiotic stress is hindered by the complexity of underlying biochemical and signaling processes on the cellular, tissue, and organ levels. Relieving the pressure on grains under heat and drought stress would require simultaneous adjustments of several enzymatic activities. Selecting specific activities and how to change them requires a systems approach to identifying the bottlenecks in the entire pathway. Thus far, multiple knowledge gaps complicate interpreting the available omics data, for example, changes in gene transcription or specific enzymatic activities in the total extracts in the context of the reduction of starch accumulation in grains. An apparent challenge is the non‐linearity of cross‐talk between different signaling components. For example, the inhibitory effect of the ABA/SnRK pathway on starch biosynthesis contrasts with the potentially beneficial role of trehalose‐6‐phosphate signaling in starch accumulation (Figure 2b). Resolving these contradictions requires amalgamating metabolic and signaling models under control and stress conditions using comprehensive experimental data.

The second challenge is the complexity of the transcriptional responses to heat and drought stress within gene families encoding components of the starch biosynthesis network (Figure 2). It is plausible that the variability of the primary structure within the gene families generates diversity in the thermal kinetic window of enzymes. Functional characterization of gene family members using reverse genetics and gene editing, taken together with information about the thermal kinetic window, will help to identify which gene family members are better suited for higher temperatures for pathway engineering. It will also be critical to measure with higher accuracy how stress perturbs different reactions in the starch biosynthesis pathway using metabolic labeling in the wild‐type and knockout backgrounds (Box 2).

Box 2 **Open questions**

1. What are the roles of trehalose‐6‐phosphate signaling in wheat grain development under normal and stress conditions?
2. What are the roles of AREB/ABF in wheat grains under stress?
3. How to enhance protein thermostability to protect yield and quality under heat and drought stresses?
4. How do abiotic stresses affect the transcription and localization of the sugar transporter in different organs?
5. Cell‐type‐specific gene expression information of developing wheat grain is missing.

### A better understanding of ABA signaling in developing grains under stress

ABA triggers adaptive responses to abiotic stresses, such as heat and drought, by influencing the expression of stress‐responsive genes. However, the signaling mechanisms and molecular pathways through which ABA mediates responses to combined stresses remain unclear. Under drought conditions, elevated ABA levels cause stomatal closure, reducing transpiration and conserving water. The roles and mechanisms of AREB/ABF in wheat grains under stress are less understood. Elucidating the regulation of these elements could reveal how ABA affects starch accumulation and stress responses.

Additionally, understanding the interplay between ABA and SnRKs for the regulation of nutrient remobilization from source to sink tissues and starch accumulation in grains is crucial. Modulating ABA levels or signaling pathways to balance nutrient remobilization with starch synthesis can be achieved via genetics without compromising the plants' ability to respond to stresses. During heat stress, the role of ABA in activating protective mechanisms, including heat shock proteins, remains speculative and requires more concrete experimental evidence.

### The opportunities for the trehalose‐6‐phosphate pathway

Under elevated temperatures, the secondary metabolite trehalose functions as an osmolyte in safeguarding a diverse array of cellular processes, whereas trehalose‐6‐phosphate acts in signaling. Trehalose‐6‐phosphate signaling could contribute to carbon allocation in developing seeds under stress through regulation of starch accumulation, such as sustaining the activity of AGPase (Figure 2b). Direct approaches for sustaining the activity of AGPase could prove challenging, considering the complexity of the activation mechanisms. Importantly, trehalose‐6‐phosphate levels correlate with sucrose levels within leaves, suggesting a role in monitoring sugar availability and influencing shifts in resource allocation by inhibiting SnRKs and promoting starch biosynthesis (Figueroa & Lunn, 2016). Other effectors could include enzymes and pathways related to seed development and stress responses. Given that seed production is the primary purpose of wheat cultivation, understanding the role of trehalose‐6‐phosphate signaling during seed development under normal and stress conditions is critical.

The level of trehalose‐6‐phosphate under stress can be sustained by reducing the activity of the TPP. Although knockdown *TPP* gene expression might seem promising, this approach could be inefficient as TPP contributes to heat and drought tolerance in cereal and non‐cereal crops (Lin et al., 2019; Nuccio et al., 2015). A better alternative could be tweaking the level of *TPP* expression. As *TPP* transcription is activated by the ABF, partially suppressing this regulatory loop by reducing the affinity of ABFs for the promoters of the *TPP* gene could lead to an increase in trehalose‐6‐phosphate levels. Another approach could be increasing the activity of TPS by inserting a heat‐inducible motif into the promoter region of *TPS* genes such as the *DREB2A* or *HSFA1* (Li et al., 2024; Zhou et al., 2020).

Achieving desired outcomes in this direction would benefit from a better understanding of the trehalose‐6‐phosphate metabolism. As a starting point, systematic measurements of trehalose‐6‐phosphate content in seeds under stress conditions should be undertaken. Second, developing techniques for determining downstream effectors of trehalose‐6‐phosphate is necessary. Third, determining whether the trehalose‐6‐phosphate signaling is cell autonomous or non‐autonomous is essential. Fourth, characterizing the balance between ABF‐ and bZIP‐dependent activation of *TPP* using gene editing is important. Fifth, tracking trehalose‐6‐phosphate metabolism under normal and stress conditions in the wild‐type and mutant plants using ^13^C‐labeled precursors UDP‐glucose or glucose‐6‐phosphate (G6P). The ^13^C‐UDP‐glucose labeling would inform on the contribution of UDP‐glucose to T6P synthesis. Feeding plants 13C‐glucose would inform on the whole‐plant trehalose‐6‐phosphate flux. Comparing labeled vs. unlabeled fractions can reveal T6P turnover and metabolic shifts during heat and drought stresses.

### Harnessing the potential of enzymatic thermostability

Heat and drought stresses affect the activity of enzymes, such as sucrose synthase and invertase, leading to the reduction in starch accumulation in grain. Enhancing the thermal tolerance of these enzymes emerges as a potential strategy for improving plant performance under abiotic stresses. However, further research is needed to explore the enzymology and metabolomics of wheat grain filling under these stressors to compile a list of candidate enzymes for enhancing thermal tolerance.

The enhancement of protein thermal stability can be achieved through various approaches. One is safeguarding the folding of enzymes by increasing the expression of heat shock proteins. Second, modifying the primary structure for enhancing the enzyme's substrate selectivity and specificity. For instance, altering amino acids in the flexible loops of *Escherichia coli* transketolase has been demonstrated to enhance enzyme thermostability (Jahromi et al., 2011). Innovative efforts have also involved inserting bacterial proteins to reroute chloroplast glycolate to glycerate, effectively reducing photorespiration and directly enhancing photosynthesis efficacy (South et al., 2019). However, specific studies focused on enhancing enzymatic activity and stability in plants under heat and drought conditions are limited. Utilizing advanced biotechnology to develop abiotic stress‐resistant varieties by enhancing protein thermostability could safeguard crop yields under heat stress.

### Conservation and diversity of stress responses

On the physiological and biochemical levels, all components of the starch biosynthesis pathway are conserved across crops. For example, ABA levels increase by 10‐fold in flag leaves and by 6‐fold in seeds of barley under drought stress (Seiler et al., 2011). In rice and maize, greater ABA levels under moderate drought correlated with enhancing sink activity, starch biosynthesis, and grain filling (Qin et al., 2013; Wang et al., 2015). However, genetic complexity remains an obstacle on the way to exploiting these processes. For example, of 41 known ABA synthesis genes in barley, 19 were regulated by terminal drought (Seiler et al., 2011). Plastid‐localized enzymes (*ZEP2*, *NCED2*, and *CCD3*) were upregulated in both leaves and seeds, while genes encoding cytosolic enzymes SDR, MCSU, and AO showed different expression patterns between leaves and seeds (Seiler et al., 2011).

In the case of *SnRK* genes, the rice gene family comprises 47 members including three *SnRK1*, ten *SnRK2*, and 34 *SnRK3* (Son & Park, 2023), the maize family of 60 genes includes four *SnRK1*, 14 *SnRK2*, and 42 *SnRK3* (Feng et al., 2022), and the wheat family of 149 genes including splice variants is made of 21 *SnRK1*, 38 *SnRK2*, and 127 *SnRK3* (Jiang et al., 2022). A similar trend applies to enzymes: the rice genome encodes 11 *TPS* and 10 *TPP* genes, whereas the wheat genome encodes 12 *TPS* and 31 *TPP* genes (Islam et al., 2021; Kerbler et al., 2023; Xie et al., 2015; Zang et al., 2011).

Similarly, SWEETs overall similar responses to stress in various crops are associated with the activation of different members of the gene family. For example, drought stress caused the up‐regulation of four cotton *SWEET* genes (*GhSWEET 5, 20, 49*, and *50*) and heat stress caused the up‐regulation of *GhSWEET4*, *5*, *10e*, *49*, and *50* (Li et al., 2018; Zhao et al., 2018). Furthermore, drought or heat stress caused the up‐regulation of *MdSWEET17* in apple (Lu, Sun, et al., 2019), *CaSWEET1‐like4* in chickpea (Bhaskarla et al., 2020), *OsSWEET13* and *15* in rice (Mathan et al., 2020), *BnSWEET9‐2*, *10‐3*, *12*, *13‐2*, and *14* in rapeseed (Jian et al., 2016), and *BrSWEET11* in *Brassica rapa* (Wei et al., 2019). However, the transcriptional responses of *SUT*s seem to be less consistent. Soybean (*GmSUT1*), barley (*HvSUT1*), rice (*OsSUT1*), and maize (*ZmSUT1*) are up‐regulated under drought (Hou et al., 2024; Xu et al., 2018), but down‐regulated under heat stress. Knowledge about the impact of stress on protein levels and their localization in different organs will aid in engineering a more resilient sugar transport system.

Selecting enzymes for engineering stress resiliency requires information about their tissue‐specific expression pattern and responsiveness to the stress conditions. Recent advancement of single‐cell/spatial transcriptome analysis enables dissecting cell‐type‐specific gene expression as compared to a whole‐tissue RNA‐seq analysis. A temporal and spatial transcriptome of developing wheat grain makes an excellent resource for untangling the genetic complexity of starch biosynthesis pathways (Li et al., 2024).

## CONFLICT OF INTEREST STATEMENT

The authors have not declared a conflict of interest.

## Data Availability

Data sharing not applicable to this article as no datasets were generated or analysed during the current study.
